# The Vast Complexity of the Epigenetic Landscape during Neurodevelopment: An Open Frame to Understanding Brain Function

**DOI:** 10.3390/ijms19051333

**Published:** 2018-05-01

**Authors:** Ariel Ernesto Cariaga-Martínez, Kilian Jesús Gutiérrez, Raúl Alelú-Paz

**Affiliations:** Laboratory for Neuroscience of Mental Disorders Elena Pessino, Canis Majoris Foundation, Madrid Scientific Park, 28049 Madrid, Spain; acariaga@canismajoris.es (A.E.C.-M.); kgutierrez@canismajoris.es (K.J.G.)

**Keywords:** epigenetic, development, neuroepigenome, DNA methylation, histone modifications, brain

## Abstract

Development is a well-defined stage-to-stage process that allows the coordination and maintenance of the structure and function of cells and their progenitors, in a complete organism embedded in an environment that, in turn, will shape cellular responses to external stimuli. Epigenetic mechanisms comprise a group of process that regulate genetic expression without changing the DNA sequence, and they contribute to the necessary plasticity of individuals to face a constantly changing medium. These mechanisms act in conjunction with genetic pools and their correct interactions will be crucial to zygote formation, embryo development, and brain tissue organization. In this work, we will summarize the main findings related to DNA methylation and histone modifications in embryonic stem cells and throughout early development phases. Furthermore, we will critically outline some key observations on how epigenetic mechanisms influence the rest of the developmental process and how long its footprint is extended from fecundation to adulthood.

## 1. Introduction

Epigenetics is a term that was coined by Waddington in 1940. During his initials works, he observed that phenotypes could be permanently established in the population’s gene pool, by action of environmental factors. Despite the role of the environment, alleles responsible for these phenotypes already exist in the population although not in the “right order” to suppose an advantage for the carriers. He correctly reasoned that genetic variability and the environment could interact and define a cell’s fate [1]. Decades later, between 1980 and 2000, methylation and histone modifications were more extensively described, and the term epigenetics adopted its current definition.

Epigenetics includes several mechanisms implied in gene expression regulation. Its hallmark is the absence of DNA sequence modification. In addition, epigenetic mechanisms are characterized by their sustainability in time despite the absence of their triggering event and its sensibility to environmental influence. It is still under discussion to what extent these changes can be transferred to the next generation, but regardless of how these marks could be inherited, epigenetics rose as a sort of “orchestra conductor” of genetic expression [2]. This tempting simile might lead to inaccurate explanations that permeate into the basic understanding of neurodevelopment, behavior studies, or mental disorders. Maybe, the possibility of quickly relating the environment to genetic data, as a kind of bridge between these apparently separate areas, could be influential in generating a naïve vision of how epigenetics really works and its implications in subject development, health, or disease proneness. To overcome this issue, current scientific efforts are directed at understanding the complete universe of interactions between epigenetic mechanisms, cellular pathways, and environmental influences, giving rise to the concept of the Epigenome [3], or, more precisely, the Neuroepigenome, when we refer to the nervous system [4], as a more comprehensive point of view, that gathers basic and clinical data instead of compartmentalized studies. 

Unfortunately, epigenetics is not able to explain as much as we would like. There are several terms (e.g., “correlation”, “association”, or “biomarkers”, among others, that frequently appear in the titles of scientific papers) used in conjunction with epigenetic observations that could act as confounders in this new frontier multidisciplinary approach. To develop a fully integrative picture, it is key to understand the specific biological changes and their putative outputs taking into consideration the genetic pool and environmental stimuli. This need is evident in complex processes such as the development of cell types, tissues, or complete organisms. On the other hand, epigenetics itself is complex and undergoes radical changes throughout an individual’s lifetime, beginning with zygote formation and gamete production.

In this work, we will cover the main epigenetic mechanisms and their changes from stem cells to adult tissue, highlighting some points with regard to brain cells. After that, we will summarize findings regarding the role of epigenetics and their implications in the developmental process itself, from gamete generation to zygote formation, and to early life stages and adulthood. In this review, it is our objective to give a concise, critical, and realistic view of how epigenetics contributes to the developmental process and how it may extend its influence throughout a lifetime. 

## 2. Starting from Scratch: Is DNA Methylation a Stable Mark?

In a non-refined search at Pubmed (https://www.ncbi.nlm.nih.gov/pubmed), the number of papers related to “DNA methylation” (as MeSH terms) has increased from around 450 in 1996 to 3600 in 2016, indicating the exceptional growing interest in this mechanism throughout these last two decades. This interest arises mainly because of the implications of DNA methylation in cancer development [5], as well as neuropsychiatric diseases such as Fragile X syndrome, where hyper-methylation at the Fragile X Mental Retardation-1 (*FMR-1*) gene is associated with intellectual disability [6].

Properly, DNA methylation refers to the methylation of the fifth carbon of cytosine to generate 5-methylcytosine (5mC). This modification mainly occurs at cytosines (C) located next to a guanine (G) residue, generating the so-called CpG sites. Sequences with a high representation of CpG sites are known as “CpG islands”. More precisely, CpG islands are 200 bp sequences with a C + G content of at least 50% and a ratio of observed to expected C + G of 0.6 [5]. This definition comprises around 1% of the genome sequence, with a major percentage corresponding to gene promoters. In this sense, DNA methylation was extensively assessed at those sites, but also at the so-called “CpG shores”, i.e., sequences with a high percentage of C + G, located near CpG islands (around 2 Kb), which show methylation patterns distinctive to certain tissues and species. However, with the advent of more refined techniques (e.g., Epigenome-wide Association Studies hereon EWAS), methylation was observed along gene bodies (including 3’ untranslated regions, exons and introns) and intergenic spaces. 

How then, could a presumably stable mark be able to interact with an environment in constant change? In human adult cells, around 70% of the genome is methylated, while unmethylated regions are mainly represented by gene promoters, hence the methylation patterns themselves tend to be stable in adult tissues [7]. Although methylation is chemically and genetically stable, it may be a substrate of refined and subtle regulations that lead to specific biological outputs, according to cell environment changes. 

DNA methylation is catalyzed by DNA methyl-transferases (DNMT) that add methyl groups from donor compounds to cytosines. There are three enzymes in charge of maintaining and generating de novo methylation and a non-catalytic isoform. DNMT1 recognizes hemimethylated sequences, and it is key to maintaining the methylation pattern during DNA replication. DNMT3a y DNMT3b catalyzes the transference of methyl donors to cytosines, so they have an active role in de novo methylation. Its catalytic activity could be enhanced by the scaffolding activity of DNMT3L, the enzymatically inactive isoform [8].

On the other hand, 5mC could be oxidized by the ten-eleven translocation enzymes 1, 2, and 3 (TET1-3) to generate 5-hydroximethylcitosine (5hmC) in vitro and in vivo [9]. This “fifth” base (or “sixth” if we consider 5mC as a different one [10]) may be understood as a new layer of information content of the genome, or as a demethylation intermediary. In either case, its roles are under intense research. 

**Figure 1:**
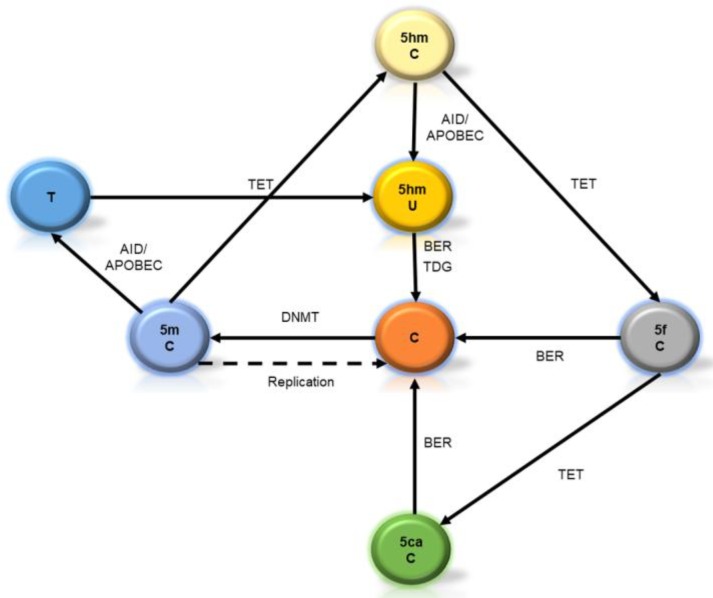
Pathways in the biogenesis of cytosine methylation in its intermediaries. C = Cytosine. 5mC = 5-methylcytosine. 5hmC = 5-hydroxymethycytosine. 5fC = 5-formilcytosine. 5caC = 5-carboxylcytosine. 5hmU = 5-hydroxymethyluracyl. T = Thymine. DNMT = DNA methyltransferases. TDG = Thymine DNA glycosylase. BER = Base excision repair system. AID = activation induced deaminase. APOBEC = Apolipoprotein B mRNA editing enzyme, catalytic polypeptide.

Although demethylation was previously thought to be only a passive process (mainly carried on during fertilization and during primordial germ cell (PGC) stage [11]), oxidation of 5hmC (catalyzed by TET enzymes, too) will progress to other intermediaries as 5-formylcytosine and 5-carboxylcytosine. These oxidized residues will be diluted in a replication-dependent manner or will be erased by the base-excision repair system (BER), leading to the generation of a non-methylated cytosine [12]. A summary of these pathways is shown in Figure 1.

From a developmental point of view, 5hmC is abundant in mouse embryonic stem cell (ES) genomes (in comparison to adult cells) and its content is positively correlated with actively transcribed genes [13]. In these cells, 5hmC was enriched at gene bodies, but to a lesser degree than at gene bodies from adult tissues [14]. Also, 5hmC seems to be instrumental for maintaining cellular self-renewal, maybe by its regulator role at the *NANOG* promoter [15]. 

In fetal brains, 5hmC enrichment was also related to actively transcribed genes, although 5hmC content is lower when compared to adult brains, but the enrichment of 5mhC at inaccessible genomic regions was interpreted as a “pre-mark” to generate unmethylated sites in later developmental stages [16].

In adult cells, 5hmC is a particularly common modification in brain tissue, constituting around 40% of all modified cytosines, and as Szulwach et al. (2011) also demonstrated, along development, 5hmC genomic content is progressively enriched in the cerebellum and hippocampus [14]. In mice, from postnatal day 7 to adulthood, 5hmC content had a 4-fold increase in the cerebellum and a 2-fold increase in the hippocampus. Also, 5hmC enrichment was observed in the actively transcribed genes of the brain, although its relevance and functions are correlated to cell types in nervous tissues [17]. Even more, 5hmC was enriched in the exon-intron boundaries, suggesting a possible role in the regulation of splicing mechanisms [18]. 

The complete picture of 5hmC functions and implications is far from established, however the aforementioned data indicate that complexity arises from solely cytosine methylation to an intricate pathway that includes several intermediaries whose functions are not completely understood. This is a vast, fertile, and unexplored field to take into account to avoid falling into a simplistic view of methylation as a mechanism that acts as a fixed mark regarding gene expression.

### Non-CG Methylation

Another type of modification was also described, i.e., non-CG methylation. This modification refers to methylation in contexts different from CpG (CpX where X may be A, T, or C). Methylation of these other residues is also dependent on DNMTs, but given its higher affinity to CpG residues, non-CG methylation is spatially tied to CpG methylation. Although non-CG methylation is relatively independent of DNMT1, it requires DNMT3L and is linked to the surrounding CpG methylation [19]. 

Non-CG methylation content and functions are cell- and specie-specific and vary along development stages. In mice ES cells, higher levels of non-CG methylation were observed in highly transcribed genes, but it seems that this mark is lost along developmental and differentiation stages. As such, in ES cells, around 75% of the total methylation is located at mCpG but it was also observed around 20% at mCpA, 4% at mCpT, and 1% at mCpC. However, in the tissue DNA, around 93% of the total methylation was observed at mCpG, 4% at mCpA, 1% at mCpT, and 1% at mCpC [20]. 

On the other hand, Ziller et al. (2011) assessed levels of non-CG methylation in different embryonic human stem cells, finding percentages that range around 10–12% of methylation (with regard to the total base number), and they observed that deletion of DNMT3a was related to a global reduction of non-CG methylation. They also confirmed that in human ES cells, the mCpA methylation event is not decoupled from mCpG [21]. Although Ziller et al. did not find impairment of pluripotency by reducing the non-CG levels, Butcher et al. (2016) observed that non-CG methylation content reduction was indeed associated with an impaired differentiation capacity of pluripotent stem cells toward endodermal lineages [22]. However, it is not clear how the differentiation process is regulated by this mark.

Also, regarding human brain tissue, Lister et al. [16] demonstrated that this modification is negligible in the human fetal brain, but relatively frequent (meaning around 1.3–1.5% of total methylation) in adult the frontal cortex, with a slight diminishment through aging. According to its findings, in brain tissue, non-CG methylation levels would be inversely correlated to generated transcripts, acting as an indicator of expressed genes. However, Guo et al. (2014) demonstrated that non-CG methylation represents around 25% of the total methylation of mice neurons from the dentate gyrus, being enriched in regions of low CpG content, and absent at protein/DNA interaction sites. In these adult cells, the content of non-CG methylation was also inversely correlated with gene expression [23].

Regarding other human adult tissues, non-CG methylation is extremely low, and varies according to cell types. In the aforementioned work, Ziller et al. (2011) also assessed levels of non-CG methylation in different human cells and tissues and find that, while 66% of citosynes are methylated (with regard to its total number), only around 1% (or minus) of A, C, or T are methylated in comparison to its total number. These percentages were also similar in other tissues as blood, rectal mucosa, or skeletal muscle cells [21].

A possible explanation to these different percentages of non-CG methylation between ES cells and neurons was assayed by Lee et al. (2017) [24]. They observed that DNMT3a and DNMT3b are differentially expressed in these cell types and that each methylate cytosines in different contexts, generating specific patterns. For instance, in ES cells DNMT3b can specifically interact with modified histones and generate a hyper-methylation of CAG sequences of actively transcribed gene bodies of development-related genes. For their part, Guo et al. (2014) demonstrated that non-CG methylation is established de novo during neuronal maturation and requires DNMT3a for active maintenance in post-mitotic neurons [23]. Although these are initial data, they seem to indicate that a poised activity of DNMTs is central in generating a specific non-CG landscape.

**Figure 2:**
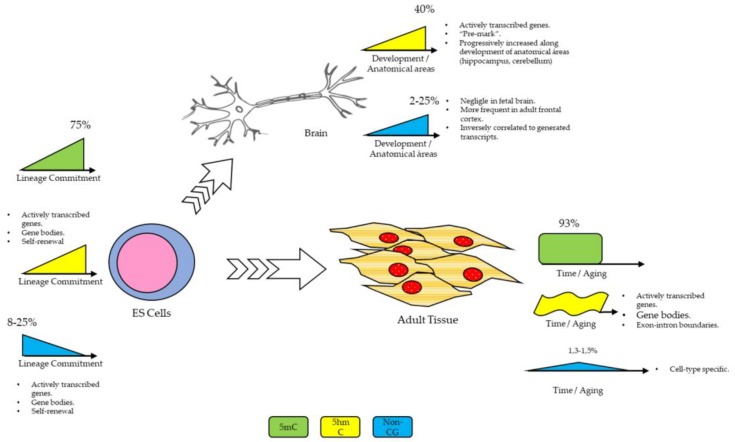
Summary of DNA methylation characteristics for cell types throughout development. Please see text for further details.

Taken together, the research indicates that the methylation landscape of the brain (either from the ES cell stage and throughout its development or as an adult tissue) is far more complex than just CpG or promoter methylation. Different intermediaries themselves can affect gene expression and add layers of complexity to the scenario. These layers also provide the cell with the adequate plasticity to generate an adaptable response to the internal and external environment. A summary of the points discussed in this section is given in Figure 2.

## 3. Histone Post-Translational Modifications: An Unstable Mark?

Interactions between genes and environment ultimately rely on the transduction of signals from a changing medium to the cell nucleus. This information undergoes several filtering processes and is finely regulated, and an adaptive cellular response is eventually released. This process is mainly directed by the protein machinery inside the cell that finally gains physical access to genes. Chromatin is a condensed status of mammal genome and its fundamental units are called nucleosomes. These are formed by a protein octamer, where two subunits of four histones (H2A, H2B, H3, and H4) form the core, while another histone (H1) separates and adjusts the complexes to facilitate the organization of DNA strands into more condensed forms. DNA is wrapped around this core, so genetic information accessibility is tightly regulated [25]. 

Histones have amino acid residues that can undergo post-translational modifications (PTM). These residues constitute the so-called “histones tails”, and once modified, they may be responsible for a plethora of biological outputs. More than 10 different types of PTMs have been described, including phosphorylation, acetylation, and methylation [26]. Even more, some modifications include additions of 1, 2, or 3 modifier groups (as methylation and acetylation), leading to differential cellular responses. In general, different PTMs are related to different biological outputs, even with opposite functions, and could be located in the same (*cis*) or in other (*trans*) histones, interacting to fine-tune the cell response [27], by “opening” and “closing” the three-dimensional conformation of chromatin, leading to regulation of the accessibility to genetic information, which leads to interactions with other epigenetic mechanisms that will finally regulate gene expression [28]. 

It is also possible that different PTMs, located at different histone molecules, generate a combinatorial pattern of biological outputs, which are currently under research to unveil their actual implications for genetic regulation [29,30,31]. This combinatorial pattern adds a layer of complexity whose implications for the final cell response to environmental stimuli, is far from established. This layer may be extensively modified along developmental stages, so in order to keep a straightforward report, hereon we will review only the more consolidated data obtained about PTMs and their currently known implications for the developmental process.

To correctly understand some concepts of the next section, we here indicate a technical point. A well-known approach for studying PTMs is by using a technique called chromatin immunoprecipitation (ChIP). In a routinely applied approach, proteins and DNA are cross-linked by chemical reaction, tying together all the protein complexes that are interacting with a specific DNA sequence in a time point. After that, complexes of DNA and protein are fragmented, and by using specific antibodies to PTMs of interest, complexes are separated. By polymerase chain reaction, it can be evaluated that the fragment of DNA is bound to the PTM. Beyond regulation of the electrostatic charge, mediated by modification of amino acid residues and its actual implication in opening and closing of chromatin structures [32], ChIP techniques popularized the notation of PTMs as “active” or “repressive” marks, taking into consideration whether marks are associated with facilitating the transcription process or machinery to proceed or not [33]. 

### Role of Histone Modifications in ES Cells: Bivalent Domains

One of the most studied PTMs are lysine acetylation and methylation of histones 3 and 4 tails. Histone H3K4me3 (trimethylation of lysine 4 at histone 3), H3K9Ac, and H3K27Ac (acetylation of either lysine 9 or 27 at histone 3) are classically accepted “activation” marks, while H3K27me3, H3K9me3, and H4K20me3 are “repressive” marks [34], among others. These marks act as recognition points for specific adaptor proteins that, in turn, will promote the recruitment of the rest of the transcription machinery. The same is said for “repressive” marks that (in general) physically affect this recruitment. To shed light on this intricate crosstalk, the terms writers, erasers, and readers are common when referring to enzymes or enzymatic complexes related to modifying or deleting histones’ PTMs or to interacting with methylation marks. 

Intriguingly, in ES cells, it was observed that “activator” PTM marks and “repressive” PTM marks were enriched at similar levels as promoters and/or the enhancer sequences of selected genes. It was demonstrated that H3K4me3 might be established in the presence of H3K27me3 at gene promoters usually related to developmental transcription factors, such as Oct4, Nanog, or Sox2 [35]. This phenomenon allows researchers to coin the term “bivalent domains” and it was observed in several thousands of other genes, mainly related to developmentally key transcription factors. In general, genes associated with bivalent marks are silenced, but lose the H3K4me3 mark after differentiation. Along differentiation and after also losing the H3K27me3, genes become “active” and are associated with increased levels of H3K4me1, H3K9me1, and H4K20me1 [36]. 

What is the role of these “bivalent domains”? As Berstein et al. (2006) indicated, these bivalent domains may keep silenced (or at least poised) certain developmental genes in ES cells, but the bivalent characteristic of the histone PTM allows them to be potentially activated accordingly to the triggered developmental program [35]. In this sense, Lien et al. (2011) demonstrated that bivalent domains are not frequently observed in adult stem cells after lineage commitment, where differentiation-related genes only showed repressive marks as H3K27me3. This observation supports the fact that the “poised” state can be a proper mechanism of ES cells that assures the adequate lineage commitment [37].

An interesting point was that bivalent domains were correlated to the methylation status of the underlying sequence in ES cells, while this correlation was lost along differentiation, leading to the proposal that embryonic state stability relies more on its DNA methylation landscape, while differentiated state stability relies more on histone PTMs [35]. 

**Figure 3:**
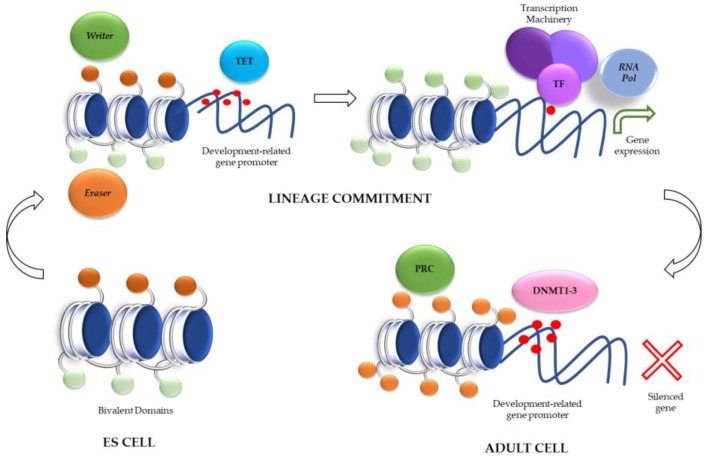
The presence of bivalent domains in ES cells is essential for maintaining a poised state. After lineage commitment, several histone erasers and writers will change the histone PTM in concordance with TET enzymes that will modify the DNA methylation status (shown as red dots). This plethora of epigenetic changes will also contribute to the expression of development-related genes. In an adult cell, repressive complexes and DNA hyper-methylation will lead to a “closed” chromatin state, avoiding the expression of development-related genes, and keeping the tissue’s identity.

Now, we can attempt to generate an extremely reduced and preliminary model of how histone PTMs mark interactions during development. In ES cells, the presence of bivalent domains confers the cell plasticity to respond to its environment and to trigger the adequate development program. After lineage commitment, a new development program promotes chromatin remodeling. In doing this, TET enzymes should remove the methylation of DNA while eraser enzymes (such as histones demethylases) must delete repressive marks from histones. Even more, other writer enzymes, such as histone acetylases, must add activation marks that in turn allow the recruitment of RNA polymerase II and the transcriptional activation of genes adequate to the triggered differentiation program. However, in adult cells the methylation landscape is different with silenced gene promoters, and with repressed genes related to differentiation programs, by action of Polycomb-Repressive Complexes (PRC). This complex oversees depositing of the silencing mark H3K27me3 and triggers a cascade that includes the recruitment of eraser enzymes (as histones deacetylases) that act on activation marks as acetylated lysines. As a result, chromatin reached a highly compact state and gene repression (Figure 3).

Finally, although bivalent domains are key to understanding the developmental process of ES cells, other early stem cells, as trophoblastic stem cells or extraembryonic endoderm stem cells, show a different pattern. In mice extraembryonic cells, bivalent domains are not frequent and show lower levels of H3K27me3 regarding embryonic cells, while H3K9me3 mark is key for repressing genes for these cells [38]. These observations indicate that during early development, there exists a lineage-dependent pattern of histone PTMs that will lead to specific biological outputs to embryonic or to extraembryonic cells. In fact, the presence of bivalent domains is tightly and quickly regulated, as observed by Liu et al. (2016) by using mice pre-implantation embryos, where bivalent domains were also infrequent and unstable. They demonstrated that in as early a developmental process as the two-cell stage, “activating” and “repressing” marks already show some “preferences”. For instance, some activation marks, such as H3K4me3, are related consistently to transcription start sites while repression marks, such as H3K27me3, are a more dynamic feature [39].

Along Section 2 and Section 3, we give a brief view of the main epigenetic mechanisms and their differences and commonalities during cellular development. There exist another epigenetic mechanisms mediated by small and long non-coding RNA. However, research on their implications in development is still in an early phase. Therefore, in the next section we will give a concise review of some crucial facts regarding this mechanism, without aiming to be exhaustive, given the amount of data generated after developing sequencing techniques, but to highlight the complexity of epigenetic mechanisms deployed over the course of neurodevelopment.

## 4. A Single-Strand Contribution: Epigenetic Mechanisms Mediated by Non-Coding RNA

Besides DNA and histone modifications, a third epigenetic mechanism, mediated by non-coding RNA, plays a major role throughout neurodevelopment. As its name indicates, non-coding RNA are transcribed RNA molecules that are not translated into proteins. These molecules comprise a heterogeneous group of long non-coding RNA (lnRNA, with a length of around 200 or more nucleotides) and small non-coding RNA (with a length of around 20 nucleotides). Among the latter are found microRNA (miRNA) and PIWI-interacting RNA (piRNA), which show specific metabolic pathways of biogenesis and functions [40,41]. These molecules are directed to regulate gene expression either by degradation of messenger RNA (mRNA) or by its contributions to the remodeling of the chromatin state [42]. Although researchers already draw a detailed view of these types of regulator RNA, current findings are only scratching the surface of their complexity [43]. Even more, this third mechanism shows extensive relationships to others as DNA methylation and histone modifications [44,45,46]. Thus, in this section we will review the main findings regarding this topic, with a focus on the roles of miRNAs in neurodevelopment.

In animal models, the deletion of genes related to miRNA biogenesis and metabolism leads to impaired neurodevelopment. In this sense, inactivation of DICER (a key enzyme for miRNA biogenesis) leads to progressive neurodegeneration, either in Purkinje cells [47] or in dopaminergic neurons [48]. Even more, the direct deletion of miRNAs itself is also deleterious to brain development. For instance, miRNA-9 inhibits the expression of the orphan nuclear receptor tailless (also known as TLX). This receptor is crucial to keeping neural stem cells in a proliferative state while remaining self-renewable [49]. In advanced stages of neurodevelopment, miRNA-9 is also implied in controling the timing of progenitor differentiation [50,51]. Also, other miRNA are implied in keeping the pluripotent state. For instance, the interactions of the miRNA-290–295 family with factors such as OCT4, Sox2, and NanoG, allow ES cells to maintain an undifferentiated state [52]. Even more, *OCT4* is directly regulated by miRNA, and this factor is able to repress the expression of miRNA-145 in pluripotent cells, while the miRNA contributes to OCT4 degradation in differentiated cells in a negative feedback loop. In neurons, upregulation of miRNA-145 is kept by miRNA-29b expression [53]. 

During neurogenesis, and after lineage commitment, some miRNAs are upregulated, while others are downregulated, generating a spatial-temporal map. For instance, in mice, neural progenitors throughout embryonic days 11 to 13, miRNA-183 is downregulated given its targets comprise several proteins related to neuronal differentiation, while miRNA-92 is upregulated given its target are the cell cycle’s related proteins [54]. 

Until complete and functional cell differentiation, miRNAs show a differential expression regulating other cellular functions such as migration. In this sense, miRNA-132 expression is critical for neuron migration and axon growth [55] while a miRNA-37–410 cluster also contributes to regulating this function. Even more, this cluster (conformed by 38 miRNAs) is located in a key imprinted region that only expresses maternal alleles [56]. Several miRNAs of this cluster have the same target, *N*-Cadherin, an adhesion molecule with crucial functions during the migration of neurons toward the cortical plate [57].

In adult brains, miRNAs are still implied in several cellular functions. For instance, miRNA-19 was implied in the migration of newborn neurons in the hippocampus [58] while miRNA-219 was implied in brain plasticity, given its capacity to regulate glutamate signaling by targeting calcium/camodulin-dependent protein kinase II gamma subunit [59]. Even more, throughout aging, miRNA content is downregulated [60], while others are differentially expressed [61]. For a more comprehensive review, please refer to [62]. 

Finally, and in addition to all the aforementioned complexity, it was also demonstrated that brain mesenchimal cells are able to deliver external miRNA to neural stem cells or progenitors leading to cell differentiation. The biological implications of this observation are largely unknown [63].

Considering that current techniques, such as Next-Generation Sequencing and RNA-Sequencing, are shedding light over the role of miRNA in neurodevelopment, it is expected that a clear increase in our understanding of this mechanism is in the near future. As a non-exhaustive summary, Table 1 collects some facts regarding miRNAs and their roles in neurodevelopment. 

As we indicate, research regarding this mechanism is frantic, but we are still far from completely understanding its global implications to neurodevelopment. Thus, in the next section, we will be focused on how DNA methylation and histone PTMs conform and define a vast and complex landscape in early development that extends throughout the lifetime.

## 5. Epigenetics of Early Development

Throughout the developmental process, the epigenetic landscape is remodeled at several stages. In this section, we will discuss some crucial epigenetic events during early embryo formation to understand its role in further development. 

We have seen that epigenetic marks are plastic enough to let the cell respond to a constantly changing environment. This plasticity is clearly observed when a differentiated nucleus is transplanted to an enucleated oocyte, where the nucleus can recover its totipotency and the offspring is fully developed [75]. However, groups of genes show a “molecular memory” regarding its parental origin. This asymmetry phenomenon is called “imprinting” and is inherited after fertilization, generating functional differences to parental genomes along development [76]. The “imprinting” mechanism also allows researcher to understand why oocytes are not totipotent cells. Given that these cells carry only a group of imprinted genes, oocytes require genes that are not “imprinted” in a paternal cell to be fully active and correctly trigger developmental programs [77]. These groups of “imprinted” genes are also markedly reluctant to epigenetic changes and their expressions remain stable. We include this term to study it as another layer of complexity, given some phases of imprinted gene expression, which are either necessary for development or implicated in disease onset, that are regulated by epigenetic mechanisms. However, we will discuss it as an annex to the general developmental process. 

### 5.1. Epigenetic Remodelling on the Zygote

The first step to understanding this complex process is fertilization and zygote formation. In mice, the asymmetry of imprinted genes is maintained for around 5 hours after fertilization. During this time, the paternal genome interacts with factors present at the oocyte’s cytoplasm and undergoes a complete demethylation, while the maternal genome undergoes a de novo methylation [78,79,80]. After pronuclear membrane formation, some proteins are translocated from oocyte cytoplasm to the pronucleus as EZH2 (Enhancer of Zeste 2 Polycomb repressive complex 2 subunit, a core protein for PRC) that form a complex with histone deacetylases and can repress genetic expression. In fact, Wu et al. (2014) showed that decreased EZH2 levels are correlated with the progressive demethylation of H3K27me3, thus allowing the expression of developmentally important transcription factors [81]. Other enzymes, such as DNMT1o, a form of DNMT1 located at the oocyte, are translocated out of the nucleus, allowing demethylation to occur [82]. 

Although this “first wave” of epigenetic landscape remodeling is radical, imprinted genes are able to resist it, and several epigenetic mechanisms that are active to maintain the imprinting were described. For instance, DNMT1o is translocated again into the nucleus at the eight-cell embryo stage, so it could promote parental imprinting preservation [83,84,85]. Although it was indicated that DNMT1 is solely sufficient for imprinting preservation in pre-implanted embryos [86], some other factors are also important, such as PGC7 (also known as Stella or Dppa3), which binds to H3K9me2 and blocks TET-3 activity on the maternal genome and on the imprinted loci of the paternal genome [87,88]. In addition, Kap1/TRIM28 is a transcriptional repressor found bound to the methylated allele of all known “imprinting control regions” (ICR), which are chromosomal regions that contain several genes acting in coordination to regulate the final expression of imprinted genes [89]. This boundary also depends on the KRüppel Associated Box (KRAB) domain zinc finger protein ZFP57 and eventually generates an epigenetic remodeler complex that interacts with histone deacetylases and methyltransferases with crucial roles for maintaining the methylation imprint after fertilization [90,91].

### 5.2. Epigenetic Remodelling on Gametes and Somatic Tissues

In parallel, the imprinting process for genes of gametes begins around day 6.5 post-fertilization, when primordial germ cells (PGC) are generated from epiblast cells. Around day 9.5, PGC migrate to the genital ridge and extensive epigenetic changes happen, leading to a complete erasure of imprinting marks as well as DNA methylation, and a concomitant activation of chromosome X that was silenced in female cells. It seems that the epigenetic erasure does not differ between male and female cells and the beginning of new imprints will occur subsequently during gametogenesis, and primarily during oogenesis [92]. With regard to spermiogenesis, histones will be replaced by protamines (and other histone variants) in a new and complete remodeling of the epigenetic landscape of paternal cells [93]. 

The new imprinting process will affect gene expression and there are several examples of interest for further research. For instance, mice embryo lethality, because of chromosome 12 distal region deletions, was attributed to the alteration in the dosage of imprinted genes. This region consists of multiple maternally expressed non-coding RNAs and several paternally expressed protein-coding genes whose balance is maintained by epigenetic mechanisms. It was observed that paternal alleles located at this region are hyper-methylated while being hypo-methylated on the maternal allele. Even more, some regions only inherit paternal methylation marks from the germline [94]. After losing this equilibrium, embryo lethality arises [95]. 

With regard to brain tissue, several diseases are related to imprinting failures. Although it is not the focus of this review, an example disease is the Prader-Willi syndrome, which is characterized by general hypotony and gradual morbid obesity development. Patients also show infertility as well as cognitive impairment [96]. This syndrome occurs when paternally imprinted genes at chromosomal region 15q11.2-q13 are lost. This chromosomal region contains an imprinting control region, with several genes that act in coordination [97]. In mice, Brant et al. (2014) observed that paternally-inherited deletion also increased methylation levels at promoters of paternally-expressed genes while maternally-inherited deletions did not yield any change to methylation status [98]. These findings, among others, indicate that beyond established genetic causes, epigenetic landscape remodeling and imprinting are key events with clear implications in brain developmental stages with a footprint that extends throughout adulthood, influencing the psychosocial and behavioral development of individuals. However, more research is needed to generate a complete framework to fully understand the role of epigenetic mechanisms during the imprinting process in human zygotes and embryos. 

## 6. Early Stress and Postnatal Environmental Influences on the Brain: An Epigenetic Answer to Long-Term Effects?

As we indicated in the last section, after fecundation and zygote formation, the epigenetic landscape is profoundly changed. However, this is not the end of epigenetic remodeling, as this process will also be carried out throughout embryonic and fetal life. Even more, it is under research as to what extent the epigenetic remodeling is also going on during adult life and its implications in disease development or proneness. 

Several environment factors such as lifestyle [64], viral infections [99], smoking [100], chronic diseases [101], or maternal stress [102] were assessed as major events responsible for affecting gene expression through epigenetic mechanisms, and presumably leading to developmental and neuropsychiatric diseases in progeny. In this sense, placenta is a key frontier for understanding the relationship of maternal inner medium’s and embryo/fetus’ gene expression changes mediated by epigenetics [103]. For instance, Paquette et al. (2016), by using a methylation array, observed that changes of placental tissue methylation were related to newborn behavior, showing that changes in infant arousal, attention, lethargy, and quality of movement were related to methylation changes at specific sites. Some of these methylation changes, such as those found in the *ANKRD11* and *FHIT* genes, showed p-values lower than 10^−7^ in association with infant attention [104]. 

Several papers describe the putative relationship between maternal stress and future psychopathologies. In general, stress responses are related to glucocorticoids metabolism, so this pathway may be a first step in understanding the role of stressors over epigenetic mechanisms. For instance, Monk et al. (2016) observed that pregnancy perceived stress was correlated with changes to methylation levels of the *HSD11B2* gene (that encodes an enzyme related to glucocorticoid inactivation) in the placental tissues [105]. The glucocorticoid receptor itself, encoded by the *NR3C1* gene, is a recurrent target in studies investigating the relationship between stress, epigenetics, and developmental impairment. In this sense, Filiberto et al. (2011) showed a correlation between *NR3C1* methylation in placental tissue and birth weight [106]. Also, Bromer et al. (2013) observed that methylation changes in the *NR3C1* gene were correlated with infantile quality of movement and attention [107]. Turecky and Meany (2016), in a recent systematic review, indicated that early-life adversity is correlated to *NR3C1* methylation in 89% of studies with human samples (data gathered from 40 reports with animal and human data) [108]. Even more, in individuals that report early trauma, reduced levels of promoter methylation of this gene were observed, and these levels were correlated with depression, anxiety, and drug-abuse disorders in adulthood [109].

After birth, and during puberty and adulthood, literature is vast regarding the putative effects of environmental injuries to epigenetic mechanisms and their consequences. In fact, the long-term effects of early stressors on human wellbeing are still under research. There are well-known results from studies of Dutch famine survivors and its consequences to progeny growth or metabolism [110]. This early exposition also persistently affected the epigenetic mechanisms of these individuals. As indicated by the fact that some imprinted genes, such as *IGF2*, still show lower methylation levels, even decades after famine exposition, when compared to control siblings [111]. In general, it was observed that prenatal famine exposure is able to affect the fetus’ epigenetic landscape, leading to a risk factor to future growth and the metabolic activities of newborns [112].

It is also tempting to find an epigenetic link to those possible environmental stimuli that effect mental disorder development. For instance, Vokojevic et al. (2014), by assessing genocide survivors, tried to find a relationship between the risk of suffering post-traumatic stress disorder and changes to the methylation levels of *NR3C1* gene [113]. Similarly, Yehuda et al. (2015) observed lower levels of methylation to this gene in the blood samples of war veterans [114]. Although interesting, there are several handicaps in these types of epigenetic approaches. Firstly, results should be assessed according to initial samples because the epigenetic signature is specific to tissues, and even anatomical regions [115]. Secondly, we only have descriptive results, and so we do not know if the methylation changes are a cause or a consequence. Furthermore, methylation changes occur throughout the life stages, such as aging, without disease conditions [116]. Although some methylation changes are persistent enough to possibly work as biomarkers of exposure [117], they are not always related to disease development, so it is necessary to clarify this confusion. In general, the results and extrapolations made from epigenetics to behavior are still in their infancy. It is our responsibility not to add confounder variables and to perform exhaustive and critical statistical analyses to unravel the actual contributions of epigenetics along an individual’s lifetime.

## 7. Future Perspectives

Development is a process that begins with two cells to form a zygote and lasts until the subjects’ death where, during this time, individuals will be interacting with a constantly changing environment. Facing this reality with a fixed and relatively stable genetic background will lead to a premature termination. Thus, several epigenetic mechanisms contribute to the necessary plasticity to ensure the proper adaptation of organisms. Given its refinement, these mechanisms add a further layer of complexity, leading scientists to intricate theoretical and methodological problems such as sample collection, model usage, and manipulation of huge amounts of information, generated by high throughput techniques. If we hope to get a truly, fully global view of the role of epigenetics throughout development, and its implications in brain function, it will be germane that these handicaps should be rigorously taken into account.

Non-invasive samples are important for basic research given that the genetic framework does not change among tissues, so it is highly probable that genetic epidemiology studies from a blood sample may be reproducible from saliva sample. However, epigenetic mechanisms respond to the environment, and not only to the external environment but to the internal medium. Cells from mucosa have many functions related to its physiology and could (or more probably, not) share epigenetic mechanisms with brain tissue. In this sense, it is tempting for scientists to obtain several types of samples to study epigenetic mechanisms in surrogate tissues. However, this type of approach adds “noise” to real facts, and results should be assessed according to initial samples because epigenetic signatures are a property of tissues and even specific anatomical regions [115]. Some statistical tools could be applied in order to interpret data obtained from surrogate tissues [118], but what we get may not represent the biological reality of development [119]. Furthermore, this point is also important for understanding when researchers develop meta-analyses, given the prestige and scientific power of these [120]. In future works, it will be extremely useful to evaluate the process of hypotheses generation to obtain a critical view of the actual value of data gotten from surrogate tissues, beyond the use of statistical tools and other possibilities. In fact, the scientific process begins without any sample collection but with an idea that becomes a realistic hypothesis.

For their part, animal models are a useful tool for understanding the role of epigenetics in development, mainly during early phases [121]. However, we should be careful not to directly extrapolate results from models. In fact, currently it is not clear as to what extension the data obtained from mice could be transferred to a human epigenetic context [122]. As we indicated throughout this review, several interactions between epigenetic mechanisms depend on the underlying sequence or are tightly regulated in time. On the other hand, biology is embedded in a complex context, so frequently a single change may generate cascades with combinatorial effects that usually are not completely considered [123].

Regarding this last point, it is possible to believe that having more epigenetic data will be more “informative” for scientific purposes. For instance, Genomic-wide Association Studies (GWAS) were the pioneer assays that revealed the genetic influence of multi-causal diseases such as cancer. However, it was not until EWAS development that researchers were able to evaluate the environmental component. Currently, it is extremely easy to assess around 1 million points of methylation on a chip. This generates huge amounts of data that require an extreme and critically careful statistical approach to be applied, provided that variables, such as sample selection, were correctly controlled. Unfortunately, we know that data will “tell the truth” if scientists “torture” them enough [124]. Although getting data for “biomarkers or biological indicators” is legitimate, we need to remember that the prestige of our profession relies on the correct application of the scientific method that, among other phases, asks us to generate adequate and realistic hypotheses. 

Brain function is still one of the most unknown mysteries of biology. Its extreme plasticity and its capacity to elicit responses to complex stimuli have caught the attention of scientists for centuries. Neuroepigenomics arose as an integrative approach whose results may generate a more complete answer to the informative gaps that still exist regarding its development. However, epigenetic landscapes show far more complexity than the classical view of “promoters hypo-methylation or hyper-methylation” as a sole cause of gene expression. Furthermore, current findings indicate that “activator” or “repressor” histone PTM constitutes a diffuse mark, with several functions under research. A complex set of interactions between epigenetic, environmental, and genetic factors is ultimately responsible for appropriate gene expression or inhibition, while the study of combinatorial effects of such interactions is still in its infancy. Even more, this intricate scenario is substantially differential throughout the developmental process. Thus, to untangle all this variability, it is our responsibility to avoid adding useless data and “noise” by assigning a strong engagement to hypothesis formulation, considering the data origin and applying a critical statistical point of view.

## Figures and Tables

**Table 1:** Selected neural progenitor-enriched and brain-enriched miRNA and their known contributions to neurodevelopment.

miRNA	Known Function	Reference
miRNA-9	Neural progenitor proliferation and maintaining of self-renewal state.	[64]
	Determination of neuron fate.	
miRNA-124	Promotes neuronal differentiation and maturation.	[65,66]
miRNA-128	Neuronal migration and plasticity.	[67]
miRNA-137	Promotes differentiation in neural stem cells from ventricular zones (embryonic mice brains) and subventricular zones (adult mice brains).	[68,69]
miRNA-132	Roles in brain plasticity and memory.	[70]
MiRNA-145	Inhibits expression of developmental factors (as Oct4).	[71]
miRNA-200	Inhibit differentiation of neurodermal precursors.	[72]
miRNA-219	Promotes oligodendrocyte differentiation.	[73]
miRNA-290/295 cluster	Promoter pluripotency and cell cycle phase distribution of ES cells.	[74]

## Abbreviations

MeSH	Medical Subject Headings
5mC	5-Methylcytosine
EWAS	Epigenome-Wide Association Studies
DNMT	DNA methyl-transferases
5hmC	5-Hydroximethylcitosine
BER	Base-excision Repair System
ES	Embryonic Stem Cells
PTM	Post translational Modifications
ChIP	Chromatin Immunoprecipitation
PRC	Polycomb-Repressive Complexes
EZH2	Enhancer of zeste 2 polycomb repressive complex 2 subunit
lnRNA	Long non-coding RNA
miRNA	MicroRNA
PGC	Primordial germ cells
